# Impact of intermittent preventive treatment of malaria in pregnancy with dihydroartemisinin-piperaquine versus sulfadoxine-pyrimethamine on the incidence of malaria in infancy: a randomized controlled trial

**DOI:** 10.1186/s12916-020-01675-x

**Published:** 2020-08-10

**Authors:** Abel Kakuru, Prasanna Jagannathan, Richard Kajubi, Teddy Ochieng, Harriet Ochokoru, Miriam Nakalembe, Tamara D. Clark, Theodore Ruel, Sarah G. Staedke, Daniel Chandramohan, Diane V. Havlir, Moses R. Kamya, Grant Dorsey

**Affiliations:** 1 London School of Hygiene and Tropical Medicine, London, UK; 2 Infectious Diseases Research Collaboration, Kampala, Uganda; 3 Department of Medicine, Stanford University, Stanford, USA; 4 Department of Obstetrics and Gynaecology, Makerere University College of Health Sciences, Kampala, Uganda; 5 Department of Medicine, University of California, San Francisco, USA; 6 Department of Paediatrics, University of California, San Francisco, USA; 7 School of Medicine, Makerere University College of Health Sciences, Kampala, Uganda

**Keywords:** Malaria, Intermittent preventive treatment, Pregnancy, Sulfadoxine-pyrimethamine, Dihydroartemisinin-piperaquine, Infants

## Abstract

**Background:**

Intermittent preventive treatment of malaria during pregnancy (IPTp) with dihydroartemisinin-piperaquine (DP) significantly reduces the burden of malaria during pregnancy compared to sulfadoxine-pyrimethamine (SP), the current standard of care, but its impact on the incidence of malaria during infancy is unknown.

**Methods:**

We conducted a double-blind randomized trial to compare the incidence of malaria during infancy among infants born to HIV-uninfected pregnant women who were randomized to monthly IPTp with either DP or SP. Infants were followed for all their medical care in a dedicated study clinic, and routine assessments were conducted every 4 weeks. At all visits, infants with fever and a positive thick blood smear were diagnosed and treated for malaria. The primary outcome was malaria incidence during the first 12 months of life. All analyses were done by modified intention to treat.

**Results:**

Of the 782 women enrolled, 687 were followed through delivery from December 9, 2016, to December 5, 2017, resulting in 678 live births: 339 born to mothers randomized to SP and 339 born to those randomized to DP. Of these, 581 infants (85.7%) were followed up to 12 months of age. Overall, the incidence of malaria was lower among infants born to mothers randomized to DP compared to SP, but the difference was not statistically significant (1.71 vs 1.98 episodes per person-year, incidence rate ratio (IRR) 0.87, 95% confidence interval (CI) 0.73–1.03, *p* = 0.11). Stratifying by infant sex, IPTp with DP was associated with a lower incidence of malaria among male infants (IRR 0.75, 95% CI 0.58–0.98, *p* = 0.03) but not female infants (IRR 0.99, 95% CI 0.79–1.24, *p* = 0.93).

**Conclusion:**

Despite the superiority of DP for IPTp, there was no evidence of a difference in malaria incidence during infancy in infants born to mothers who received DP compared to those born to mothers who received SP. Only male infants appeared to benefit from IPTp-DP suggesting that IPTp-DP may provide additional benefits beyond birth. Further research is needed to further explore the benefits of DP versus SP for IPTp on the health outcomes of infants.

**Trial registration:**

ClinicalTrials.gov, NCT02793622. Registered on June 8, 2016.

## Background

Infection with malaria parasites during pregnancy remains a major public health problem, especially in sub-Saharan Africa where transmission intensity is highest and *Plasmodium falciparum* the predominant species. In 2018, there were an estimated 39 million pregnancies in sub-Saharan Africa, of which over 11 million (29%) were exposed to *P. falciparum* [1]. The majority of women residing in malaria-endemic areas of sub-Saharan Africa are partially immune and do not develop symptoms when infected with *P. falciparum* during pregnancy. However, even in the absence of symptomatic disease, malaria in pregnancy is associated with maternal anemia and adverse birth outcomes such as low birth weight, preterm delivery, and stillbirth [2–4].

To prevent malaria in pregnancy and improve birth outcomes, the World Health Organization (WHO) recommends intermittent preventive treatment (IPTp) with sulfadoxine-pyrimethamine (SP) in pregnant women residing in areas of moderate to high malaria transmission intensity [5]. However, the effectiveness of IPTp-SP is threatened by widespread antifolate resistance resulting in failure to clear parasites and prevent new infections [6]. Recent studies have shown dihydroartemisinin-piperaquine (DP) to be a promising alternative to SP for IPTp. Compared to IPTp-SP, IPTp-DP has been shown to be much more effective at reducing the prevalence of malaria parasitemia and incidence of clinical malaria during pregnancy and reducing the risk of placental malaria at delivery [7–9]. However, despite significantly reducing the burden of malaria during pregnancy, IPTp-DP has not been shown to be superior to IPTp-SP at improving adverse birth outcomes [7–9].

Prevention of malaria during pregnancy may have additional benefits to the infant beyond the neonatal period. Studies have shown that intrauterine exposure to *P. falciparum* may negatively affect the development of antimalarial immunity in the infant [10, 11]. Indeed, several observational studies have suggested that placental malaria increases the risk of malaria during infancy [12–14]. However, these studies could not rule out the possible confounding effect of behavioral, environmental, genetic, and social-economic factors shared by the mother and her infant on the associations between exposure to malaria parasites during pregnancy and risk of malaria during infancy. A more robust method of testing this hypothesis would be to compare the impact of a highly effective regimen versus a less effective regimen for IPTp on the risk of malaria during infancy in a randomized controlled trial. However, to date, clinical trials that have evaluated the impact of IPTp on the risk of malaria during infancy have been limited by little difference in the efficacy of IPTp regimens [15, 16] or the provision of chemoprevention during infancy, in addition to IPTp [17].

To address this gap in evidence, we compared the incidence of malaria during the first year of life among infants born to HIV-uninfected pregnant women who took part in a double-blind randomized controlled trial of monthly IPTp with DP (a highly effective regimen) versus SP (a less effective regimen). During pregnancy, IPTp-DP was superior to IPTp-SP at reducing the incidence of clinical malaria and prevalence of asymptomatic parasitemia during pregnancy, and the prevalence of placental malaria at delivery [9]. Children born to mothers enrolled in this study did not receive chemoprevention during infancy.

## Methods

### Study setting and participants

This study was conducted from September 6, 2016, to December 4, 2018, in Busia district, south-eastern Uganda, an area of high malaria transmission intensity. The study was conducted in two phases: the pregnancy phase, which involved enrollment and follow-up of pregnant women through delivery, and the infancy phase, which involved follow-up of infants through 12 months of age. Details of the pregnancy phase of the study have been published elsewhere [9]. In brief, HIV-uninfected pregnant women were eligible for enrollment if they were 12–20 weeks of gestation, 16 years or older, agreed to come to the study clinic for any illness, had no history of taking IPTp-SP or any other antimalarial therapy during the current pregnancy, and provided written informed consent. Women were excluded if they had a history of serious adverse events to SP or DP. The infancy phase involved the follow-up of all live births among women enrolled in the pregnancy phase of the study.

### Study design, randomization, and masking

This was a double-blind randomized controlled trial designed to assess the impact of monthly IPTp with DP versus SP in HIV-uninfected pregnant women, on the incidence of malaria during infancy (trial registration, ClinicalTrials.gov; NCT02793622). At enrollment, pregnant women were randomly assigned in a 1:1 ratio to receive IPTp-DP or IPTp-SP. A randomization list was computer generated using permuted blocks of 4 or 8 by a staff member not directly involved in patient care. To achieve allocation concealment, sealed envelopes, each containing a treatment allocation number and treatment group assignment, were prepared following the sequence of the randomization list, prior to enrollment. Treatment allocation was done by a study pharmacist not involved in daily patient care by picking the next available sealed envelope, recording the participant’s identification number on the envelope, and opening it to reveal the assigned treatment. The treatment allocation number, the participant’s identification number, and the assigned treatment were then recorded on a treatment allocation log which was kept in a safe lockable place only accessible by the study pharmacist. Study drugs for each enrolled participant were pre-packaged by the study pharmacist and labeled with the participant’s identification number. Study drugs were administered every 4 weeks starting at 16 or 20 weeks of gestation. Each dose of DP (tablets of 40 mg of dihydroartemisinin and 320 mg of piperaquine; Duo-Cotexin, Holley-Cotec, Beijing, China) consisted of 3 tablets given once a day for 3 consecutive days. Each dose of SP (tablets of 500 mg of sulfadoxine and 25 mg of pyrimethamine; Kamsidar, Kampala Pharmaceutical Industries) consisted of 3 tablets given as a single dose. To achieve blinding, participants randomized to DP also received SP placebos, and participants randomized to SP received DP placebos every 4 weeks. The administration of all 1st daily doses of study drugs was directly observed in the study clinic. The 2nd and 3rd daily doses were dispensed to the mother for self-administration at home. Adherence to the 2nd and 3rd daily doses was assessed by self-reporting during the visits following routine visits.

### Study procedures

Study procedures for pregnant women have been previously described in detail [9]. Briefly, at enrollment, all participants received a long-lasting insecticide-treated net and underwent a standard history and physical examination. Pregnant women were encouraged to come to a dedicated study clinic any time they were ill and to attend routine visits conducted every 4 weeks for study drug dispensing and laboratory testing. Pregnant women diagnosed with symptomatic malaria detected by microscopy at any visit were treated with artemether-lumefantrine. Asymptomatic parasitemia detected during routine visits was not treated. At delivery, a standardized assessment was completed including evaluation of birth weight, gestation age based on ultrasound dating, and collection of biological specimens including placental tissue and placental blood.

Following delivery, all live births were followed up to 12 months of age. Mothers were encouraged to bring their infants to a dedicated study clinic open every day for all their medical care and were provided a transport refund. Routine assessments were conducted every 4 weeks including the collection of blood for the detection of parasites by microscopy. Infants who presented with a history of fever in the past 24 h or with a documented tympanic temperature > 38.0 °C had blood collected for a thick blood smear. If the thick blood smear was positive, infants were treated for malaria according to the Uganda Ministry of Health guidelines which consisted of artemether-lumefantrine for uncomplicated malaria and intravenous artesunate for complicated malaria. Non-malarial illnesses were treated according to the integrated management of childhood illnesses guidelines. At 12, 28, and 52 weeks of age, blood was collected for hemoglobin measurement.

### Laboratory procedures

The presence of malaria parasites in dried placental blood spots was detected by loop-mediated isothermal amplification as previously described [18]. Placental malaria by histology defined as the presence of malaria parasites or malaria pigment was detected from placental tissue as previously described [19]. Blood smears were stained with 2% Giemsa and read by experienced microscopists. A blood smear was considered negative when the examination of 100 high-power fields did not reveal asexual parasites. All blood smears were read by two independent microscopists. Blood smears with discrepant results between the first and the second readers were read by a third reader as a tiebreaker. Hemoglobin measurements were made using a portable spectrophotometer (Hemocue, Angelholm, Sweden).

### Study outcomes

The primary outcome was the incidence of malaria from birth to 12 months of age. An incident episode of malaria was defined as the presence of fever (history of fever in the past 24 h or a tympanic temperature ≥ 38.0 °C) with a positive thick blood smear not preceded by another malaria episode in the last 14 days. Secondary outcomes included time to the first episode of malaria, incidence of complicated malaria defined as an episode of malaria with danger signs (any of the following, less than 3 convulsions over 24 h, inability to sit or stand, vomiting everything, unable to breastfeed or drink) or the meeting standardized criteria for severe malaria, incidence of all-cause hospitalizations, infant mortality, incidence of non-malarial febrile illnesses, prevalence of malaria parasitemia during routine visits, and prevalence of anemia (hemoglobin < 10 g/dL) at 12, 28, and 52 weeks of age.

### Statistical analysis

To test the hypothesis that infants born to mothers randomized to IPTp-DP would have a lower incidence of malaria during the first 12 months of life compared to infants born to mothers randomized to IPTp-SP, it was estimated that the incidence of malaria in infants born to mothers randomized to IPTp-SP would be 3–5 episodes per person-year (using data from a prior study conducted in the adjacent district of Tororo [20] and a loss of 5% of follow-up time per year). With these assumptions, the study had 80% power to detect an 18–23% difference in the incidence of malaria (incidence rate ratio of 0.77–0.82) among infants born to mothers randomized to IPTp-DP compared to those born to mothers randomized to IPTp-SP with a two-sided significance level of 0.05.

Data were double entered and verified in Microsoft Access by two independent data entrants. Using Stata, version 14.2, statistical analyses were done in the modified intention-to-treat population, which included all live births followed until they reached 12 months of age or premature study withdrawal. Comparisons of simple proportions were made using the chi-square test or Fisher’s exact test. Comparisons of continuous variables were made using the *t* test. Comparisons of proportions with repeated measures were made with generalized estimating equations, with the use of log-binomial regression and robust standard errors to adjust for clustering. Comparisons of incidence measures were made using a negative binomial regression model. Incidence rate ratios (IRRs) were defined as the incidence in the IPTp-DP arm divided by the incidence in the IPTp-SP arm. Prevalence ratios were defined as the prevalence in the IPTp-DP arm divided by the prevalence in the IPTp-SP arm. Stratified analyses of our primary outcome according to infant sex and age, and maternal gravidity were planned a priori in the statistical analysis plan. The cumulative risk of the first episode of malaria was compared using a Cox proportional hazards model with the association expressed as the hazard ratio (HR). In all analyses, two-sided *p* values of < 0.05 were considered statistically significant.

## Results

### Study participants and follow-up

**Fig. 1:**
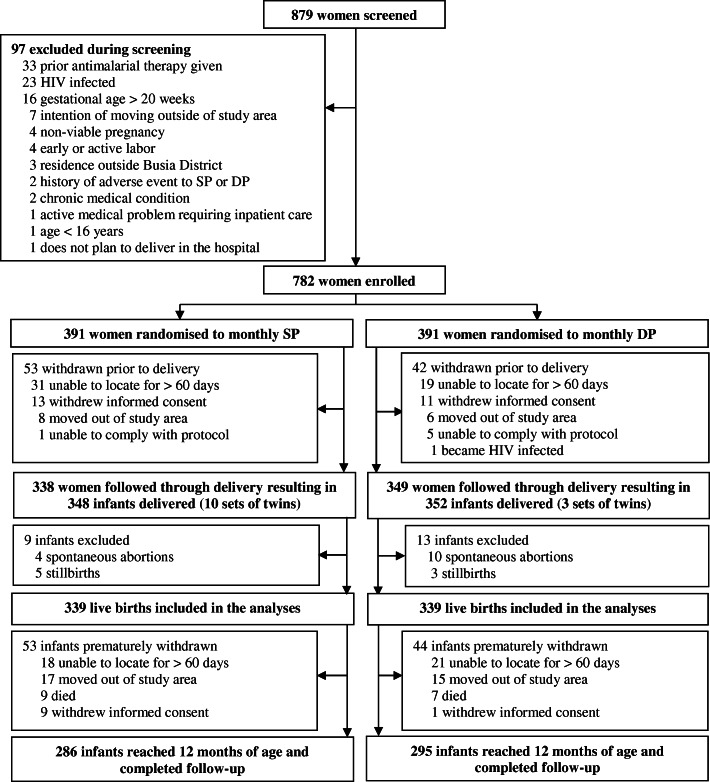
Trial profile. SP, sulfadoxine-pyrimethamine; DP, dihydroartemisinin-piperaquine

Between September 2016 and May 2017, 879 pregnant women were screened and 782 were enrolled and randomized to receive either monthly IPTp with DP (*N* = 391) or SP (*N* = 391) (Fig. 1) [9]. Among the 687 (87.9%) women who were followed through delivery, there were 678 live births from December 9, 2016, to December 5, 2017, 339 in the IPTp-DP group and 339 in the IPTp-SP group. Among the 678 live births, 581 infants (85.7%) were followed up to 12 months of age (Fig. 1). Maternal characteristics at enrollment were similar between the two groups (Table 1). Mean age at enrollment was 23.9 years, 321 (47.3%) infants were born to primigravida or secundigravida mothers, and 399 (58.8%) infants were born to mothers enrolled at 12–16 weeks of gestation while 279 (41.2%) were born to mothers enrolled at > 16–20 weeks of gestation. Three hundred fifty-three (52.1%) infants were born to mothers with parasitemia detected by microscopy at enrollment. During pregnancy, the period prevalence of parasitemia detected by microscopy during routine visits was significantly lower in mothers who received IPTp-DP (365/2324, 15.7%) compared to mothers who received IPTp-SP (828/2291, 36.1%, *p* < 0.001). Similarly, the incidence of malaria during pregnancy was significantly lower in mothers who received IPTp-DP compared to mothers who received IPTp-SP (0.09 vs 0.59 episodes per person-year, *p* < 0.001). At delivery, compared to mothers who received IPTp-SP, mothers who received IPTp-DP had a significantly lower prevalence of placental parasitemia detected by histology (28.3% vs 60.9%, *p* < 0.001, Table 1). However, at birth, there were no significant differences in the characteristics of infants born to mothers who received IPTp-DP or IPTp-SP (Table 1).

**Table 1:** Characteristics of study participants and their mothers

Characteristic	Maternal IPTp arm		*p* value
	Monthly SP (*N* = 339)	Monthly DP (*N* = 339)	
Maternal characteristics at enrollment			
Age in years, mean (SD)	23.9 (5.9)	23.9 (5.7)	0.98
Gravidity, *n* (%)			
Primigravida	86 (25.4%)	73 (21.5)	0.21
Secundigravida	72 (21.2%)	90 (26.6)	
Multigravida	181 (53.4%)	176 (51.8)	
Gestation age categories, *n* (%)			
12–16 weeks	195 (57.5%)	204 (60.2%)	0.48
> 16–20 weeks	144 (42.5%)	135 (39.8%)	
Parasite prevalence by microscopy, *n* (%)	170 (50.2%)	183 (54.0%)	0.32
Maternal characteristics during pregnancy			
Parasite prevalence by microscopy^a^, *n*/*N* (%)	828/2291 (36.1%)	365/2324 (15.7%)	< 0.001
Incidence of malaria (episodes/PPY)	0.59	0.09	< 0.001
Measures of placental malaria			
Placental blood positive by microscopy^b^, *n*/*N* (%)	29/326 (8.9%)	1/331 (0.3%)	< 0.001
Placental blood positive by LAMP^c^, *n*/*N* (%)	71/320 (22.2%)	7/329 (2.1%)	< 0.001
Positive placental histology^d^, *n*/*N* (%)	199/327 (60.9%)	93/329 (28.3%)	< 0.001
Characteristics of infants at birth			
Preterm birth, *n* (%)	27 (8.0%)	17 (5.0%)	0.12
Gestation age in weeks, mean (SD)	39.4 (1.9)	39.6 (1.6)	0.08
Low birth weight, *n* (%)	34 (10.0%)	26 (7.7%)	0.28
Birth weight in grams, mean (SD)	3052 (505)	3023 (408)	0.41
Female sex, *n* (%)	166 (49.0%)	180 (53.1%)	0.28

*DP* dihydroartemisinin-piperaquine, *IPTp* intermittent preventive treatment of malaria in pregnancy, *LAMP* loop-mediated isothermal amplification, *PPY* per person-year, *SD* standard deviation, *SP* sulfadoxine-pyrimethamine

^a^Defined as the number of positive blood smears during routine visits divided by the total number of routine blood smears

^b^Defined as the detection of parasites in the placental blood by microscopy

^c^Defined as the detection of parasites in the placental blood by LAMP

^d^Defined as the detection of parasites or pigment in the placental tissue

### Impact of IPTp with DP on incidence of malaria in infancy

**Fig. 2:**
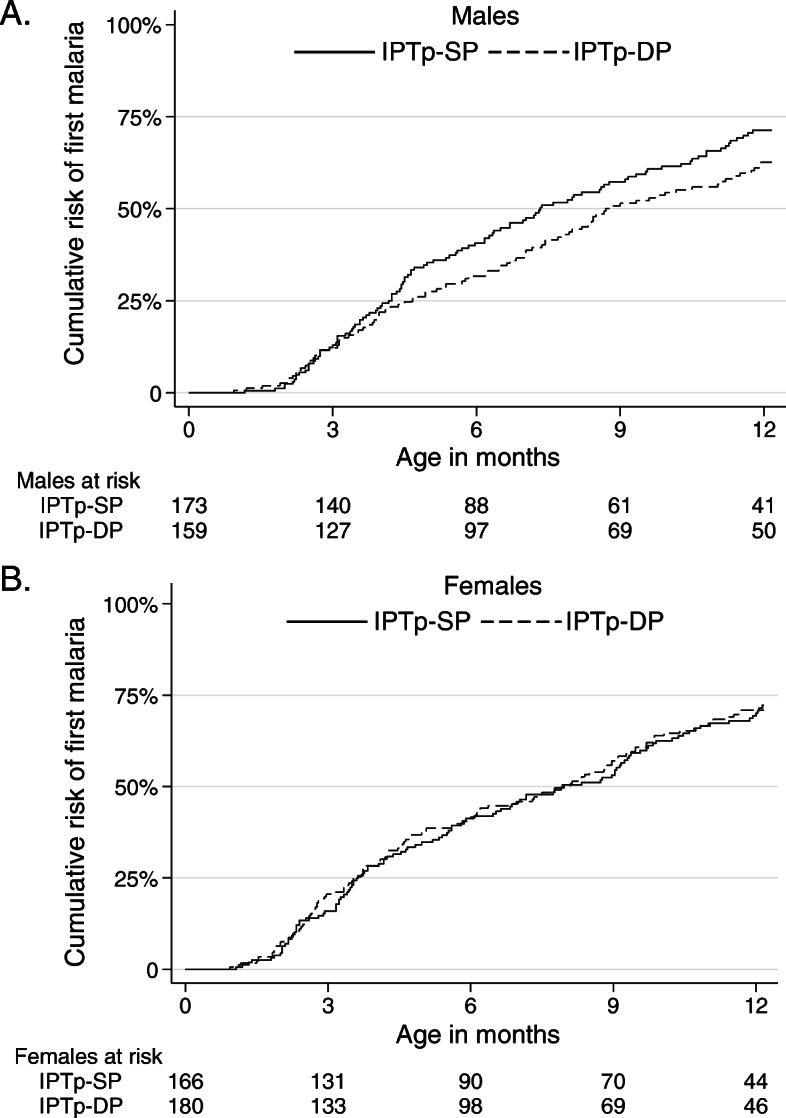
Time to the first episode of malaria stratified by infant sex. **a** Males. **b** Females. IPTp, intermittent preventive treatment of malaria in pregnancy; SP, sulfadoxine-pyrimethamine; DP, dihydroartemisinin-piperaquine

Overall, infants born into the cohort experienced 1131 episodes of malaria during 614 person-years of follow-up. The incidence of malaria was lower among infants born to mothers who received IPTp-DP (1.71 episodes per person-year) compared to infants born to mothers who received IPTp-SP (1.98 episodes per person-year), but the difference was not statistically significant (incidence rate ratio (IRR) 0.87, 95% confidence interval (CI) 0.73–1.03, *p* = 0.11; Table 2). However, the association between IPTp and the incidence of malaria in infants was modified by infant sex. Among male infants, the incidence of malaria was significantly lower among infants born to mothers who received IPTp-DP compared to those born to mothers who received IPTp-SP (IRR 0.75, 95% CI 0.58–0.98, *p* = 0.03). There was no difference in the incidence of malaria between female infants born to mothers who received IPTp-DP versus IPTp-SP (IRR 0.99, 95% CI 0.79–1.24, *p* = 0.93; Table 2). In addition, the difference in malaria incidence between the IPTp regimens among male infants was only significant between > 3 and 12 months of age (IRR 0.73, 95% CI 0.56–0.96, *p* = 0.02) but not between 0 and 3 months of age (IRR 0.89, 95% CI 0.48–1.66, *p* = 0.72). There were no differences between the IPTp regimens among female infants stratified by age (Table 2). There was no significant difference in the time to the first episode of malaria between infants born to mothers who received IPTp-DP and infants born to mothers who received IPTp-SP (hazard ratio (HR) 0.90, 95% CI 0.75–1.09, *p* = 0.30). When stratified by infant sex, male infants born to mothers who received IPTp-DP had a lower rate of the first episode of malaria compared to male infants born to mothers who received IPTp-SP, but this did not reach statistical significance (HR 0.79, 95% CI 0.60–1.05, *p* = 0.10) and there was no difference among female infants (HR 1.01, 95% CI 0.78–1.31, *p* = 0.96; Fig. 2).

**Table 2:** Impact of IPTp on the incidence of malaria during infancy stratified by sex, age, and gravidity

Strata	Maternal IPTp arm	Number of infants	Episodes of malaria	Person-years of follow-up	Incidence of malaria (PPY)	IRR (95% CI)	*p* value
All infants born alive	SP	339	602	304.4	1.98	0.87 (0.73–1.03)	0.11
	DP	339	529	309.2	1.71		
Sex							
Female infants	SP	166	283	152.5	1.86	0.99 (0.79–1.24)	0.93
	DP	180	303	164.8	1.84		
Male infants	SP	173	319	151.8	2.10	0.75 (0.58–0.98)	0.03
	DP	159	226	144.3	1.57		
Female infants stratified by age							
0–3 months of age	SP	166	30	40.1	0.75	1.16 (0.72–1.89)	0.54
	DP	180	38	43.6	0.87		
> 3–12 months of age	SP	156	253	112.5	2.25	0.97 (0.77–1.24)	0.83
	DP	168	265	121.3	2.19		
Male infants stratified by age							
0–3 months of age	SP	173	22	41.5	0.53	0.89 (0.48–1.66)	0.72
	DP	159	18	38.2	0.47		
> 3–12 months of age	SP	160	297	110.3	2.69	0.73 (0.56–0.96)	0.02
	DP	145	208	106.1	1.96		
Maternal gravidity							
1	SP	86	131	69.3	1.89	0.84 (0.58–1.20)	0.33
	DP	73	102	64.6	1.58		
2	SP	72	97	65.5	1.48	1.04 (0.72–1.49)	0.85
	DP	90	124	81.1	1.53		
> 3	SP	181	374	169.5	2.21	0.84 (0.67–1.07)	0.15
	DP	176	303	163.5	1.85		

*CI* confidence interval, *DP* dihydroartemisinin-piperaquine, *IPTp* intermittent preventive treatment of malaria in pregnancy, *IRR* incidence rate ratio, *PPY* per person-year, *SP* sulfadoxine-pyrimethamine

### Impact of IPTp with DP on incidence of complicated malaria in infancy

Overall, there were 68 episodes of complicated malaria among 59 different infants. For 60 episodes of complicated malaria, only danger signs were present while 8 episodes met the criteria for severe malaria (7 episodes of respiratory distress and 1 episode of severe anemia). The incidence of complicated malaria was lower among infants born to mothers who received IPTp-DP compared to those born to mothers who received IPTp-SP (IRR = 0.54, 95% CI 0.32–0.92, *p* = 0.02; Table 3). Again, effect modification by infant sex was observed. Male infants born to mothers who received IPTp-DP had a significantly lower incidence of complicated malaria compared to male infants born to mothers who received IPTp-SP (IRR 0.36, 95% CI 0.17–0.78, *p* = 0.01). There was no significant difference in the incidence of complicated malaria between female infants born to mothers who received IPTp-DP or IPTp-SP (IRR 0.86, 95% CI 0.40–1.87, *p* = 0.71).

**Table 3:** Secondary outcomes

Incidence measures	Maternal IPTp arm	Number of cases (incidence PPY)	IRR (95% CI)	*p* value
Complicated malaria	SP	44 (0.145)	0.54 (0.32–0.92)	0.02
	DP	24 (0.078)		
All-cause hospitalisations	SP	19 (0.062)	0.39 (0.15–1.05)	0.06
	DP	8 (0.026)		
Infant mortality	SP	9 (0.030)	0.45 (0.03–7.88)	0.59
	DP	7 (0.023)		
Non-malarial febrile illnesses	SP	1022 (3.36)	1.01 (0.91–1.12)	0.87
	DP	1047 (3.39)		
Prevalence measures^a^	Maternal IPTp arm	Prevalence (%)	PR (95% CI)	*p* value
Parasitemia^b^	SP	344/3879 (8.9%)	1.02 (0.83–1.27)	0.84
	DP	357/3933 (9.1%)		
Anemia^c^	SP	222/878 (25.3%)	0.96 (0.79–1.17)	0.70
	DP	216/892 (24.2%)		

*CI* confidence interval, *DP* dihydroartemisinin-piperaquine, *IPTp* intermittent preventive treatment of malaria in pregnancy, *IRR* incidence rate ratio, *PPY* per person-year, *PR* prevalence ratio, *SP* sulfadoxine-pyrimethamine

^a^Prevalence measures are period prevalence

^b^Proportion of blood smears with parasitemia measured routinely every 4 weeks starting at 4 weeks of age

^c^Defined as the proportion with hemoglobin < 10 g/dL measure routinely at 12, 28, and 52 weeks of age

### Impact of IPTp with DP on other secondary outcomes

The incidence of all-cause hospitalizations was lower among infants born to mothers who received IPTp-DP compared to infants born to mothers who received IPTp- SP, but this did not reach statistical significance (IRR 0.39, 95% CI 0.15–1.05, *p* = 0.06). When stratified by sex, male infants born to mothers who received IPTp-DP had a statistically significantly lower incidence of all-cause hospitalizations compared to male infants born to mothers who received IPTp-SP (IRR 0.20, 95% CI 0.05–0.82, *p* = 0.03), but there was no difference among female infants (IRR 1.01, 95% CI 0.22–4.63, *p* = 0.99). A total of 16 infants died, including 9 in the neonatal period. One infant born to a mother who received IPTp-DP died of malaria at 11 months of age. There was no significant difference in the mortality rate between infants born to mothers who received IPTp-DP or IPTp-SP (IRR 0.45, 95% CI 0.03–7.88, *p* = 0.59). The incidence of non-malarial febrile illnesses was similar among infants born to mothers who received IPTp-DP or IPTp-SP. The prevalence of parasitemia during routine visits and prevalence of anemia (hemoglobin< 10 g/dL) measured at 12, 28, and 52 weeks of age were similar among infants born to mothers who received IPTp-DP or IPTp-SP. Considering the time to the first parasitemia, there was no significant difference between infants born to mothers who received IPTp-DP and those born to mothers who received IPTp-SP (HR 0.92, 95% CI 0.76–1.10, *p* = 0.37). When stratified by infant sex, male infants born to mothers who received IPTp-DP had a slightly lower rate of first parasitemia compared to male infants born to mothers who received IPTp-SP, but this did not reach statistical significance (HR 0.81, 95% CI 0.62–1.06, *p* = 0.13) and there was no difference among female infants (HR 1.02, 95% CI 0.79–1.31, *p* = 0.87).

## Discussion

In this double-blind randomized controlled trial conducted in an area of high malaria transmission intensity, infants born to mothers randomized to IPTp-DP had a 13% lower incidence of malaria compared to infants born to mothers randomized to IPTp-SP, but the difference was not statistically significant. However, among male infants, IPTp-DP was associated with a lower incidence of malaria compared to IPTp-SP. This difference was primarily seen between > 3 and 12 months of age. Similarly, male infants born to mothers who received IPTp-DP had a lower incidence of complicated malaria and all-cause hospitalizations compared to male infants born to mothers who received IPTp-SP. In contrast, among female infants, there were no differences in the incidence of malaria, complicated malaria, and all-cause hospitalizations between those born to mothers who received IPTp-DP versus IPTp-SP. For both sexes, there were no significant associations between maternal IPTp regimen and the risk of non-malarial febrile illness or anemia during infancy.

IPTp-DP remains an attractive alternative to IPTp-SP for the prevention of malaria in HIV-uninfected pregnant women. Three randomized controlled trials conducted recently in East Africa have consistently shown that IPTp-DP is associated with significant reductions in the risk of maternal parasitemia, maternal clinical malaria, and placental malaria compared to IPTp-SP, yet none of these studies has shown significant differences in the risk of adverse birth outcomes for infants born to mothers receiving DP or SP for IPTp [7–9]. Historically, IPTp policy recommendations have been driven by evidence supporting reductions in the risk of adverse birth outcomes, specifically low birth weight, and for this reason, the WHO continues to recommend IPTp-SP [5]. However, improved protection from malaria infection during pregnancy may have longer-term effects on infant health [21]. A wealth of immunologic evidence suggests that maternal malaria infection affects the development of the fetal and infant immune system, both by altering the immune graft received by the fetus (both maternal antibodies and cells) as well as exposing the fetus to parasite antigens that may alter both the innate and adaptive immune responses [22]. Indeed, infants born to mothers with placental malaria may be at an increased risk of malaria during infancy, and it would be important to know if more effective IPTp regimens could reduce the risk of malaria in infants [13, 14].

In this study, although there was no overall evidence of a reduced malaria incidence in infants born to mothers receiving IPTp-DP versus IPTp-SP, our overall findings suggest that IPTp-DP may be associated with a moderately lower incidence of malaria in infants. A recently published systematic review identified three randomized controlled trials which evaluated the impact of different IPTp regimens on malaria during infancy [23]. There were no significant differences in the incidence of malaria among infants born to mothers randomized to IPTp with mefloquine versus IPTp-SP [16], intermittent screening and treatment with AL versus IPTp-SP [15], or IPTp-SP versus placebo [14]. However, these studies were limited by the failure of the alternative (non-SP) regimen to significantly reduce the risk of placental malaria. In a randomized controlled trial from Uganda, IPTp-DP was found to significantly reduce the risk of placental malaria compared to IPTp-SP [8]. But, surprisingly, the incidence of malaria during the first 2 years of life in infants born into this cohort was higher in infants born to mothers who received monthly IPTp-DP compared to those born to mothers who received IPTp-SP given every 2 months [17]. However, in that study, all infants were also given DP every 3 months, and the association between IPTp-DP and increased malaria during infancy was only observed in females. This finding could largely be explained by the fact that female infants with in utero exposure to DP had lower piperaquine levels after receiving DP during infancy, which is strongly predictive of malaria risk [24]. In contrast, among male infants, in utero exposure to DP was not associated with piperaquine levels during infancy [17]. Furthermore, male infants born to mothers randomized to IPTp-DP had a trend towards a lower incidence of malaria compared to male infants born to mothers who received IPTp-SP, a similar finding to that reported in here (aIRR 0.66, 95% CI 0.25–1.75) [17].

In the present study, IPTp-DP was associated with a lower incidence of malaria, a lower incidence of complicated malaria, and a lower incidence of all-cause hospitalizations during infancy compared to IPTp-SP, but only among male infants. Although the precise mechanism by which infant sex modifies the relationship between maternal IPTp, maternal malaria infection, and infant malaria risk remains uncertain, there is a growing body of evidence of sex-based differences in susceptibility to infectious diseases in infants [25, 26]. Male infants have been found to have an increased predisposition to more frequent and more severe manifestations of infectious diseases than females [26]. Similarly, several adverse pregnancy outcomes, including stillbirth, are more common in males than in females [27]. This suggests that in utero fetal exposures may have more severe consequences for male infants than female infants [28]. Several potential mechanisms for these sex-based differences have been described. These include genetic differences attributable to the heterogeneity of expression of X chromosome encoded genes [29], sex-dependent differences in glucocorticoid receptor expression and fetal-placental responsivity to cortisol [30], and sex-specific differences in neonatal and infant immune responses to Toll-like receptor ligands and induction of regulatory T cell populations [25, 31]. Given the reduced risk of malaria among male infants whose mothers received IPTp-DP compared to those whose mothers received IPTp-SP, we hypothesize that effective prevention of maternal malaria may prevent sex-specific adverse consequences of placental malaria.

Our study had some limitations. The observed incidence of malaria in infants born to mothers who received IPTp-SP (1.98 episodes per person-year) was lower than the assumed incidence of 3–5 episodes per person per year which was used for sample size estimation. This limited the power of the study to detect a significant difference between the two IPTp arms if such a difference truly existed. Also, our study was not powered to conduct stratified analyses by infant sex and age, or to test for associations between IPTp-DP and secondary outcomes such as complicated malaria and all-cause hospitalizations, which were relatively uncommon in our study. Therefore, statistically significant associations observed in stratified analyses and between IPTp-DP and secondary outcomes should be interpreted with caution. This study was conducted in an area with very high malaria transmission intensity, as at enrollment over 80% of mothers had malaria parasitemia detected by microscopy or quantitative PCR [9]. This limits the generalization of our findings to other areas with lower transmission intensity. Also, the administration of all SP doses was directly observed while only the 1st daily dose of DP was directly observed. It is possible that some mothers did not take the 2nd and 3rd doses of DP which were dispensed for self-administration at home. This could have led to an underestimation of the effect of IPTp-DP compared to SP on the incidence of malaria. Finally, we measured malaria incidence using passive surveillance which could have underestimated the true incidence of malaria if infants were treated for malaria at home or taken for care outside the study clinic. However, mothers were encouraged to bring their infants to the study clinic for care whenever they were sick and were provided a transport refund.

## Conclusion

In summary, our study findings show that in addition to reducing the burden of malaria during pregnancy and placental malaria at delivery compared to monthly IPTp-SP [9], monthly IPTp-DP was associated with a reduced incidence of malaria, complicated malaria, and a trend towards a reduced incidence of all-cause hospitalizations among male infants born to HIV-uninfected pregnant women residing in a high malaria transmission setting. Improved prevention of malaria during pregnancy may have additional benefits beyond birth. These results provide additional support for replacing SP with DP for IPTp in areas with widespread antifolate resistance. Future studies of IPTp should consider follow-up of infants beyond the neonatal period to evaluate the potential impact of IPTp on infant outcomes, stratify results based on infant sex, and evaluate the impact of IPTp on infant outcomes in settings of moderate malaria transmission intensity.

## Data Availability

Data collected for the study including individual participant data and data dictionaries defining fields in the datasets have been made available to others through a request to the Eunice Kennedy Shriver National Institute of Child Health and Human Development (NICHD) Data and Specimen Hub (DASH): https://dash.nichd.nih.gov/Resource/Tutorial. Data can be accessed through the NICHD-DASH website, https://dash.nichd.nih.gov/Study/20027, following user registration and a research data request process. The NICHD DASH Data Access Committee reviews all requests to determine that a requester’s proposed use of the data is scientifically and ethically appropriate and does not conflict with constraints or informed consent limitations identified by the institutions that submitted the data.

## Abbreviations

CI: Confidence interval
DP: Dihydroartemisinin-piperaquine
HR: Hazard ratio
IPTp: Intermittent preventive treatment of malaria in pregnancy
IRR: Incident rate ratio
LAMP: Loop-mediated isothermal amplification
PR: Prevalence ratio
SP: Sulfadoxine-pyrimethamine
